# A New Compendium of Treatment

**Published:** 1912-06-08

**Authors:** 


### A NEW COMPENDIUM OF TREATMENT.

### Medical and Surgical Therapeutics of To-day.

Last week was published by Messrs. J. and A. Churchill a "System of Treatment," in four large volumes, edited by Dr. A. C. Latham and Mr. T. C. English. This important work is the joint product of a very large number of contributors: the great bulk of them are British, though a few of them hail from America and from the Continent of Europe. Many of the authors are London specialists, but there is a very fairly strong contingent of provincial representatives and also of Scottish, Irish, and foreign clinicians. The four volumes are splendidly printed and well illustrated; and the feat of issuing the set complete on one and the same day is naturally a source of legitimate pride to editors and publishers. Each volume contains about 1,100 pages, exclusive of a general index; and is published at a guinea. This ambitious compilation must have entailed upon the editors, both of them busy men, an enormous amount of labour. It is, therefore, not to be wondered at, though it is a pity, that in several instances articles have been passed which stood in need of extensive revision from the point of view of clarity of expression and literary elegance. Amongst so numerous a band of contributors it is inevitable that some should lack the gift of exposition; but the polishing up of work which is immature in this respect is one of the important functions of an editor.

In general terms it may be stated that modern treatment is exhibited fully and sanely in this "System." There are, of course, errors and omissions here and there; but before we particularise a few of them it should be understood that they are exceptional, and that an extraordinarily high general level of excellence is maintained. Indeed, it is this very consistency which throws somewhat into relief the few contributions which might have been better.

In the first two volumes, which deal chiefly with general surgical and medical subjects, there is a certain diversity of literary arrangement. Thus one writer is allowed many pages to explain his own particular technique of uniting fractures, while the surgical treatment of colitis is dismissed in barely eight pages. As an example of the "System" at its best, we may point to Mr. Ernest Lane's contribution dealing with syphilis, which gives in epitome one of the best accounts of the modern treatment of syphilis. Mr. Ernest Lane rightly lays stress on the importance, above all things, of long-continued mercurial treatment; his opinion on the arsenic compounds and the sarsaparilla treatment is expressed with caution but without dogmatism, and with it every practitioner who is not unduly prejudiced in favour of the new will concur. The main fault of the surgical section of the "System" is the abrupt manner in which many of the subjects are handled. In some cases the text is severely abbreviated, treatment being dismissed in a line. Indeed, if one estimates modern advance in treatment solely on what is contained in some of the articles in these volumes, one obtains a gloomy impression of the impotence of medical science to deal with diseased conditions—a gloomier impression, in our opinion, than compilers of a "System" ought to give. Another fault which has to be found with some of the contributors is the lack of acquaintance with the literature which, with commendable exceptions, is too frequently displayed. There is a conspicuous absence of any attempt at systematic compilation of a bibliography at the end of each section, which is one of the best and most useful features of Allbutt's "System of Medicine." On the contrary, many articles have no reference whatever, and others merely one or two citations. Nor do all the writers appear to be acquainted either with the history of the very subject on which they write or with recent Continental literature regarding it. So well-known an operation as Maydls bladder transplantation is ascribed to an English surgeon; and an article dealing with the Bier treatment is far inferior to that on the same subject in the third volume. The indications for and against a special method are in some cases not dealt with as they should be in a clear and concise manner. Perhaps this is due to the fact that the writers who fall short in this respect are young men whose clinical experience is not great enough to enable them to differentiate between text-book statements. Certainly one recognises an enhanced practical value in the articles written by men who, like Dr. Rolleston, Dr. Spriggs, and Messrs. Armour and Lane, have had wide clinical experience and possess the gift of communicating their opinions in sound English.

The most valuable articles in the second volume are, from the surgical point of view, Mr. Legg's notes on the surgical treatment of affection of the thyroid, which would have gained immeasurably if the whole subject had been considered under one heading; Mr. Grove's article on diseases of the jaws; Mr. Hutchinson's too brief chapter on diseases of the tongue; and Mr. Corner's on abdominal injury. Mr. Mayo-Robson gives nothing new or original in his discussion of stomach and duodenal lesions; Mr. English deals with appendicitis in a stepmotherly way which does not enlarge the reader's knowledge much beyond ordinary text-book limits. In the first volume Mr. Ernest Lane's articles are obviously the best, but we must note appreciatively Mr. Armour's chapters on head injuries and cranial lesions. The important subject of the surgical treatment of paralyses is treated in a slipshod fashion which leaves out of account, apparently, much of the recent work which has been done at Graz, Bologna, and Berlin. Dr. Keith contributes an interesting chapter on asphyxia, in which he counsels the adoption of the Schäfer method of artificial respiration. It should be stated that this method has been received with far less favour abroad than in this country; and it is by no means definitely settled whether it is really the best.

The third volume is devoted to special methods of treatment, special regions, and special diseases. First of all comes an article of forty pages on anæsthetics, by Dr. Blumfeld. Into this small space he has succeeded in compressing a most astonishing quantity of well-arranged and practical information; but the inclusion of short articles on this subject in "Systems" of surgery or treatment is in principle wrong, though certainly if anything could reconcile one to it, Dr. Blumfeld's account would do it. In advising three grains of cocaine as the maximum for subcutaneous injection in producing local anæsthesia, the author seems to be involving his disciples in very considerable risk of causing severe toxic symptoms. Probably the paragraphs on spinal anæsthesia were written some time ago, for Barker's old solution is mentioned, not that which he now uses. Nor can the statement be accepted that there has been no death from status lymphaticus under ether: McCardie in 1907 collected six such cases.

The next article deals with Bier's hyperæmic treatment. Mr. H. F. Waterhouse, the author, is an enthusiastic advocate of this system of therapy, and continued to use it during the long period of neglect which preceded the boom of two or three years ago. His favour is, however, discriminating; and if the limitations which are here outlined were properly understood, there is little doubt that the method would achieve more success than it has yet done in this country. There is a not unpleasing note of personality about this article which adds weight to the teachings embodied in it. Chronic cases are more certainly benefited, Mr. Waterhouse finds, than acute ones. Pus should always be treated on the usual surgical lines; indeed, it is suggested that Bier's treatment affords a diagnostic criterion in cases of doubtful suppuration, because it at once makes the symptoms worse if pus has formed. Chilblains, amongst other lesions, are said to be rapidly relieved: this is a thing worth knowing of and experimenting with.

Follows an article on climatology which contains many interesting and arresting remarks upon this subject. Guiding principles are enunciated rather than details of individual health resorts. An attempt is made, in fact, to reduce climatology to a science; and if it cannot be said that the guidance is very definite, it is still something that it is based upon intelligible and rational principles.

Electro-therapeutics, hypnotism, light treatment, physical exercises, and massage are handled by experts in those subjects; and to hydrology (= balneology) almost fifty pages are allotted. The latter article contains a useful index of spas: but the endeavour to explain the effects of various kinds of baths on a physiological basis is frequently unconvincing. Next come a series of articles upon serum and vaccine therapy; the first is by Dr. Latham himself, and indicates how very vague and uncertain knowledge of the actions of these agents still is. Dr. Latham, it may be noted, still upholds the oral administration of vaccines, though he admits that most authorities disapprove of it.

Tropical diseases are discussed by contributors who have special knowledge of them; Drs. Daniels and Low are responsible for most of these articles, which embody the most recent researches. Diseases of the eye, ear, throat, nose, and skin occupy the remainder of the volume; these sections are all in safe hands, and the treatment advised is practically always that which is in favour with the bulk of specialists in those departments. To give one instance only, Mr. Hett describes both the guillotine and the enucleation method of dealing with chronically enlarged tonsils; and points out the cases for which these operations are respectively suitable

The fourth and last volume of the set includes obstetrics and genæcology. While many of the authors are London specialists, there are also a good many from the provinces, Scotland, and Ireland. Thus representation is afforded to different schools of obstetric practice and teaching; and occasionally divergencies of treatment are apparent, which is, of course, inevitable. Thus, Dr. Tweedy sutures a torn perineum (before expulsion of the placenta) with the patient in the lateral posture; whereas further on Dr. Lockyer advises Clover's crutch and the lithotomy posture for the same operation. Probably most clinicians will prefer Dr. Lockyer's plan. Dr. Dakin, who deals with accidental hæmorrhage and placenta prævia, is in favour of vaginal plugging in certain cases; this Irish method British obstetricians have been slow to endorse, and indeed it has been attacked quite recently; but those who have tried it in suitable cases generally form a good impression of it.

Amongst the many helpful and admirable contributions it is difficult to praise any without seeming to disparage the rest. But Dr. Gibbon's on dysmenorrhœa and on sterility deserve especial mention for broad outlook and practical common sense. Mr. Bonney's articles on diseases of the uterus are enriched with some of his extremely clever drawings from the *Text-book of Gynæcological Surgery* which he and Dr. Comyns Berkeley published a year ago. He holds very strongly that carcinoma of the cervix is the result of long continued chronic cervicitis, and that every puerperal woman should be douched antiseptically until all discharge ceases. Indeed, he goes so far as to compare the cervicitis of parous women with chronic superficial glossitis of the tongue as antecedents of malignant disease. Any distinction between "erosion" and carcinoma he regards as futile, because the former is, he says, the invariable precursor of the latter. The important subject of fibroids is excellently treated of by Dr. Comyns Berkeley, who also contributes on version, craniotomy, forceps, and similar procedures. Maxwell's intra-uterine saline douche for patients already septic from careless manipulations is not mentioned in the "general considerations," enumerated by the same author as applying to obstetric operations. Dr. Freeland's account of the management of the new-born child is another meritorious article. Finally, we may draw attention to the admirable index, which comprises all four volumes, and is printed entire at the end of each.

